# Breakfast consumption and associated factors and barriers among school-aged children

**DOI:** 10.3389/fnut.2024.1423301

**Published:** 2024-09-03

**Authors:** Zelalem Aneley, Hirut Assaye, Habitamu Mekonen, Yenewa Bewket, Embet Lake, Andualem Fentahun

**Affiliations:** ^1^Department of Human Nutrition, College of Medicine and Health Science, University of Debre Markos, Debre Markos, Ethiopia; ^2^Department of Applied Human Nutrition, Faculty of Chemical and Food Engineering, University of Bahir Dar, Bahir Dar, Ethiopia; ^3^Department of Environmental Science, College of Medicine and Health Science, University of Debre Markos, Debre Markos, Ethiopia; ^4^Department of Health Informatics, College of Medicine and health Science, University of Debre Markos, Debre Markos, Ethiopia

**Keywords:** breakfast, children, primary school, performance, breakfast consumption, cognitive

## Abstract

**Background:**

Breakfast provides the energy and nutrition we need to function at best, both mentally and physically. It is also plausible to propose that children’s general development is impacted when they skip breakfast.

**Objective:**

To assess the prevalence of breakfast consumption and associated factors and predictors among public primary school children in Debremarkos Town, Northwestern Ethiopia, 2020.

**Methods:**

A community-based cross-sectional study was conducted among school-aged children in Debremarkos, Northwest Ethiopia. A multistage random sampling technique was used to select 609 study participants. The children’s parents were interviewed using a pretested, structured questionnaire. For the rest of the analysis, SPSS version 20 was used. Logistic regression analysis was performed to assess the factors associated with breakfast consumption. Statistical significance was determined at a *p* value <0.05.

**Results:**

Out of the sampled children, 600 respondents participated in the study, for a response rate of 98.5%. The prevalence of regular breakfast consumption among school-aged children was 67.5%. Higher odds of regular breakfast consumption were found among respondents who were females (AOR = 1.72, 95% CI = 0.118–1.773), those who lived in high-income families (AOR = 7.33, 95% CI = 1.036–8.110), and those who had an educated family (AOR = 13.05, 95% CI = 0.019–13.1). However, lower odds of regular breakfast intake were found among respondents aged 9–12 years (AOR = 0.54, 95% CI = 0.369–0.79).

**Conclusion:**

Breakfast is a major health concern for school-aged children in Debremarkos city. Breakfast eating is associated with several factors; the most notable factor is being female, having a high income, and having an uneducated family. Therefore, to prevent children from skipping breakfast, stakeholders must move swiftly.

## 1 Introduction

Childhood is a critical phase and a key stage of growth and development; thus, it is important to achieve appropriate, balanced nourishment throughout this period (1). The average cerebral blood flow and oxygen utilization of children are 1.8 and 1.3 times greater than those of adults, respectively (2). Furthermore, because children require more sleep than adults, the extended overnight fasting period might deplete glycogen during the night (3). To maintain this elevated metabolic rate, a consistent supply of glucose-derived energy is necessary; hence, breakfast may be vital for providing enough energy in the morning (3).

Breakfast is the first meal of the day, and it provides the brain with the energy it requires to operate cognitively (4). Breakfast eating is considered important for children’s academic success and cognitive health, yet irregular breakfast eating is bad for a person’s psychological and cognitive development (5). Children who eat breakfast regularly are more likely to consume a diet high in dietary fiber and total carbohydrates and low in fat and cholesterol, among other beneficial nutrients (6).

Despite the benefits of breakfast, the prevalence of breakfast consumption among children and adolescents was found to be between 70% and 90% worldwide, with a lower prevalence among girls and children from lower socioeconomic backgrounds (7). In developing countries, breakfast consumption has decreased to 55% among 15-year-old and increased to 61% among 13-year-old (8). Furthermore, 2.5% of students irregularly ate breakfast every school day, while 14.0% of students irregularly ate breakfast for at least 1 day (9). According to a study conducted in Indonesia, the Philippines, and Thailand, 1–13% of children—including those in Malaysia and Singapore—irregularly eat breakfast. Among the several barriers to eating breakfast were that food was unavailable, they were not hungry, or they did not have enough time to eat breakfast (10).

A study conducted in southern Ethiopia indicated that 57.8% of people regularly eat breakfast. The most frequent barriers to eating breakfast were a loss of appetite, a shortage of food, and a lack of time to eat (11). To design and execute specific remedies and treatments, it is imperative to analyze children’s breakfast eating habits and associated factors and barriers due to the harmful consequences that irregular breakfast eating has on students’ cognition and performance. Thus, this study examined breakfast intake and associated factors and predictors among school-aged children to establish a baseline of data for lobbying and education for reform.

## 2 Materials and methods

### 2.1 Study setting and design

The study was carried out in Debremarkos city, East Gojjam zone. The settlement is located 265 km from Bahir Dar, the capital of the Amhara National Regional State, and 300 km from Addis Ababa. The town contains 17 kindergartens, 23 primary schools—15 public and eight private—two preparatory schools, 15 adult education institutions, 11 distinct colleges, and one university, according to data from the municipality’s education department. Overall, there were 7,473 public elementary school students, 3,831 of whom were female. An institution-based cross-sectional study design was employed from February 20 to April 21, 2022.

### 2.2 Population and eligibility criteria

Children aged 7–12 years who attended primary schools composed the source population, whereas children attending the selected primary schools composed the study population. Children under the age of 7 and those above the age of 12 were excluded.

### 2.3 Sample size and sampling technique

The sample size was determined using a single population proportion formula, with the following assumptions: a 95% confidence level, a 5% margin of error, and a 42% proportion of primary school children eating breakfast from a previous study conducted in the Sidama zone, southern Ethiopia (11). After multiplying by 1.5 and accounting for a 10% nonresponse rate, the total sample size was 609. A multistage sampling technique was utilized to choose the study participants. Out of a total of 15 primary schools, four were chosen at random using a lottery method. Following a proportional allocation of students to each school based on the total number of primary school students, study participants were selected from the primary schools using a systematic random sampling technique. The parameter K was determined by dividing the study population (*N*) by the desired sample size (*n*).

### 2.4 Operational definitions

Breakfast consumption: dichotomous classifications were used (≥5 days/week, <5 days/week) (12).
Regular breakfast consumption was defined as follows: when students ate breakfast for 5–7 days per week.
Irregular breakfast consumption was defined as follows: when students ate breakfast for <5 days per week.
Primary school children: children who attend primary school and are in the age category of 7–12 years as established by the ESDP (13).
School food retailer environment: availability of fast food vendors at walking distance of school (14).

### 2.5 Data collection tools and procedure

An interviewer-administered and semi structured questionnaire was used to collect the data. Socio-demographic characteristics and socioeconomic status were assessed using questions adapted from the Ethiopia Demographic and Health Survey 2012 report (15). A two-item questionnaire from a previous study was utilized to collect data on breakfast intake (12). The survey asked about breakfast intake on both weekdays and weekends. The answers vary from “irregular breakfast intake” to “regular breakfast intake.” The overall score was divided into two groups: regular breakfast intake (5–7 times per week) and irregular breakfast intake (5–7 times per week). A standardized questionnaire was utilized to gather information about the children’s entire breakfast consumption. Specifically, caretakers were asked if children had eaten breakfast on the day of the interview, how many times they had eaten breakfast in the previous week, and if they skipped breakfast and why. After the students were selected from the schools, their household address was traced in the students’ parent database. Then, the data collectors went to the children’s house to interview the parents/caretakers.

#### 2.5.1 Dietary intake

Dietary consumption data were obtained from caretakers. Using the 24-h recall method, the caretakers were asked to recall meals their children had eaten over the previous 24 h. To aid with the recall and estimation of food quantities consumed, household measurements (such as cups, ladles, and spoons) and food models were employed.

#### 2.5.2 School food retail environment

Fast-food businesses within 1 km of schools were chosen since they can walk in 10–15 min (16), making it a convenient option for children to breathe.

### 2.6 Data quality control

The questionnaire was prepared in English, translated into Amharic, and then returned to English to guarantee consistency. A pretest was conducted on approximately 5% of the sample. Supervisors and data collectors both received training. The data collection procedure was closely monitored, and the completeness of the data was assessed accordingly. A 24-h recall approach was used to collect nutritional intake data.

### 2.7 Data analysis

The data were coded, inputted, and cleaned with Epi data version 3.1. All the statistical tests were carried out using SPSS data analysis software. Descriptive statistical analysis used frequency, percentage, and *p* value to describe the study population through explanation. To investigate the association between the dependent and independent variables, bivariate analysis was used. A significant association between the dependent and independent variables was defined as a *p* value <0.05. After accounting for the potential confounding factors of other predictor variables, a multivariate analysis was performed to identify the factors associated with breakfast intake. Variables with *p* values <0.2 in the bivariate analysis were added to the multivariate model, followed by stepwise backward regression.

## 3 Results

### 3.1 Socio-demographic characteristics

The study included 600 children, for a 98.5% response rate. Regarding parents’ educational status, 33.5% of mothers and 10% of fathers were unable to read and write. The majority of parents (87.8%) were married, the majority (73%) of children’s families had <5 members, and 67% of families had a high monthly income (Table 1).

**Table 1:** Socio-demographic characteristics of the parents of the study participants in Debremarkos, Northeastern Ethiopia, 2020 (*n* = 600).

Variables	Category	Frequency	Percent
Religion	Orthodox	579	96.5
	Muslim	10	1.7
	Protestant	11	1.8
Residence	Urban	563	93.8
	Rural	37	6.2
Marital status	Married	527	87.8
	Divorced	30	5.0
	Widowed	43	7.2
Father education	Unable to read and write	60	10.0
	Read and write only	236	39.3
	Primary school	6	1.0
	Secondary school	12	2.0
	Preparatory	57	9.5
	College and above	229	38.1
Mother education	No formal education	201	33.5
	Read and write only	203	33.8
	Primary	10	1.7
	Secondary	19	3.2
	Preparatory	53	8.8
	College and above	110	18.3
Family income	<1,000 ETB	43	7.2
	1,000–2,000 ETB	155	25.8
	>2,000 ETB	402	67
Family size	≤ 5	438	73
	>5	162	27

### 3.2 Child characteristics

Out of the 600 sampled children, 583 (93.8%) of the majority of the study participants traveled to school within 30 min. The mean age of the study participants was 9.98 (± 1.76), ranging from 7 to 12 years. The majority of the study participants (563, 93.8%) were urban, 579 (96.8%) were Orthodox and 168 (28%) were grade 6 (Table 2).

**Table 2:** Characteristics of the study participants in Debremarkos, Northwestern Ethiopia, 2020 (*n* = 600).

Variables	Category	Frequency	Percent
Age	6–9 years	237	39.5
	10–12 years	363	61.5
Sex	Male	305	50.8
	Female	295	49.2
Distance from home to school	≤ 30 min	583	93.8
	>30 min	37	6.2
Living with	Both parents	492	82
	Mother only	65	11

### 3.3 School food retailer environment

Among the four sampled schools, 75% had cafeteria and all served fast foods, sweet drinks and serve sweet foods. Fast food vendors are available within a 5–10-min walking distance in 50% of schools (Table 3).

**Table 3:** School environment of study participants in Debremarkos, North West Ethiopia, 2020 (*N* = 609).

Variables	Category	Frequency	Percent
School cafeteria	Yes	3	75
	No	1	25
Cafe serve sweet foods	Yes	3	75
	No	1	25
Proximity of fast food vendors	>10 min	1	25
	5–10 min	2	50
	<5 min	1	25

### 3.4 Prevalence of breakfast consumption

**Figure 1:**
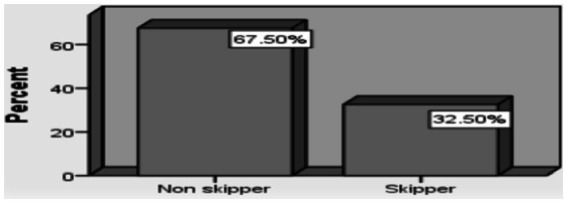
Pr breakfast consumption among school aged children.

With regard to breakfast eating, 405 (67.5%) primary school students regularly ate breakfast, and 195 (32.5%) irregularly ate breakfast (skipping three or four times per week) (Figure 1).

### 3.5 Predicators of breakfast consumption among school-aged children

**Figure 2:**
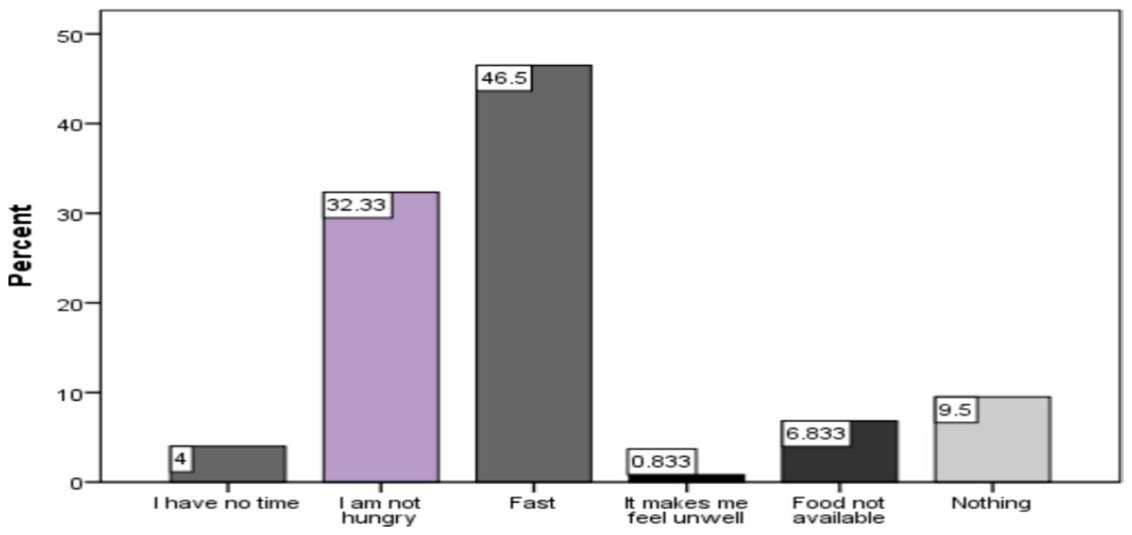
Predicators of breakfast consumption among school aged children.

Approximately 42% of the study participants reported fasting, particularly on Wednesdays and Fridays, 32.3% said they were not hungry to eat, and 0.8% said they felt ill after eating breakfast (Figure 2).

### 3.6 Typical meal for breakfast

**Figure 3:**
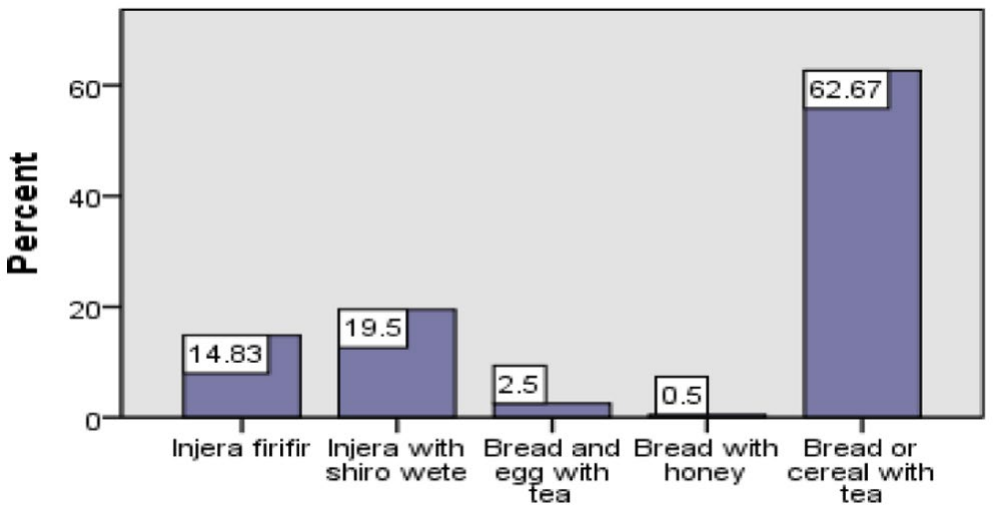
Typical meals for breakfast consumption among school aged children.

Approximately 62.67% of the students had tea with bread or cereal for breakfast, 0.5% had honey with bread, and another 2.5% had bread and eggs with tea. Most of the students had Injera with shiro wet (19.5%) and Injera firifir (15%) (Figure 3).

### 3.7 Factors associated with skipping breakfast

In the bivariate logistic regression model, variables including age, sex, marital status, living status, occupation of mother and father, education of mother and father, types of foods and drinks, distance from home to school, school cafeteria, proximity of fast food vendors, and socioeconomic status were significantly associated with the breakfast habits of school-age children (*p* value <0.25).

After adjusting for multivariate analysis, age, sex, socioeconomic status, and mother/caretaker education were significantly associated with the breakfast habits of the students. After controlling for relevant covariates, there were 1.72 females (AOR = 1.72, 95% CI = 0. 0.118, 1.773) more likely to eat breakfast every day than males. Compared with students aged 6–9 years, 46% (AOR = 0.543, 95% CI = 0.369, 0.799) of students aged 10–12 years were less likely to have breakfast every day. Furthermore, students from high-income homes were 11 times more likely to have breakfast than students from low-income families were [AOR = 13.077, 95% CI (1.007, 13.826)]. Children with educated mothers (college or higher) are 12 times more likely to eat breakfast on a regular basis than are children with noneducated mothers [AOR = 12.118, 95% CI (0.015, 12.953)] (Table 4).

**Table 4:** Breakfast consumption behaviors among school-aged children, Debremarkos city, Northeast Ethiopia, 2020 (*n* = 600).

Breakfast consumption variable	Category	Frequency	Percent
Breakfast consumption	Regular breakfast consumer	405	67.5
	Irregular breakfast consumer	195	32.5
Typical meal for breakfast	Bread with tea	376	62.67
	Injera firfir	89	14.83
	Injera with shiro wet	117	19.5
	Bread with honey	3	0.5
	Bread and egg with tea	15	2.5

## 4 Discussion

This cross-sectional study examined breakfast consumption and its associated factors and predictors among primary school children in Debremarkos. It was observed that the children consumed a high amount of regular breakfast. Regular breakfast consumption was favorably associated with being aged 6–9 years and coming from a high-income, educated family. However, being male was a negative predictor. The main predictors of regular breakfast eating are feeling unwell, not being hungry, not having food available, religious reasons (for example, fasting), and a lack of time.

The current study revealed that 67.5% of respondents ate breakfast on a regular basis. This percentage is greater than that reported in a study performed in China, which indicated that 61.5% of school-age children eat breakfast daily (17). A similar study indicated that more than 61.9% of study participants responded that they ate breakfast regularly (18). This value is greater than those reported in Sidama, Ethiopia, and Rives State, Nigeria (19, 20). This finding was lower than that of studies carried out in the Netherlands, Canada, Saudi Arabia, and Ghana, where the prevalence was 95% and 72.7%, respectively (21–24).

This study also revealed that gender, age, family education, and family income all have an impact on regular breakfast consumption. The present study revealed that respondent gender predicted regular breakfast consumption, which is consistent with the findings of numerous prior studies indicating that females are more likely to consume regular breakfast (6, 25). This suggests that more emphasis should be placed on encouraging regular breakfast consumption among male children.

Moreover, the age of the respondents predicts whether or not they regularly ate breakfast, which is in line with the findings of Heo et al. (26), where the age of the respondents significantly influenced their intake of breakfast. This may suggest that 6–9-year-old children have better control by their family to regularly eat breakfast. This could also be attributed to a greater understanding of the importance of breakfast, which has made families more likely to encourage children to eat breakfast on a regular basis.

The lack of time being a barrier to regular breakfast consumption matches prior studies that also found that lack of time in the morning frequently restricted regular breakfast intake (27). This is consistent with the fact that due to a lack of time, many students are unable to eat breakfast at home and are thus more inclined to rely on various street foods, such as fried foods, which are less expensive and less time-consuming.

Children with well-educated parents or guardians were more likely to eat breakfast frequently than were those without (AOR = 12.1, 95% CI 0.015, 12.95). Different studies have reported comparable findings (28–30). One possible justification is that noneducated parents are less likely to grasp the cognitive and physical benefits of their children eating breakfast on a regular basis. That is, parental education programs have the potential to influence children’s eating habits and enhance the healthy behaviors they engage in throughout their lives. Earlier research indicated feasible solutions, such as the provision of free breakfast in schools and regular teaching for parents about healthy eating in children to promote regular breakfast eating (31–33).

## 5 Conclusion and recommendation

Breakfast eating was relatively high in this study. Breakfast consumption has been linked to cognitive health and performance. Parental illiteracy, parental income, children’s age, and gender were all significant factors. As a result, it is preferable to implement and focus on children by spreading awareness about the benefits of daily breakfast, particularly among noneducated parents. It is also vital to develop school health and nutrition initiatives, as well as partners with key stakeholders such as childcare providers and other professionals who work with young children and their families, to improve eating habits. Furthermore, future studies should examine the quality of breakfast through a follow-up study.

### 5.1 Limitations

The current study was limited to measuring the quality of breakfast, which may improve children’s breakfast consumption. Another limitation of this study is that it does not show the nutritional status of the children.

## Data Availability

The original contributions presented in the study are included in the article/supplementary material; further inquiries can be directed to the corresponding author.
